# Physical Capacity Changes Following a Virtual Reality-Assisted Exercise Program in Institutionalized Older Adults: A Pilot Intervention Study

**DOI:** 10.3390/healthcare14162472

**Published:** 2026-08-10

**Authors:** Rosa Areiza, Fermina Vasquez Osorio, Leily Montoya-Alvarez, Edgar Rodriguez-Sepulveda, Lizt Perez, Ana Sánchez Pérez, Sergey Camilo Echeverri Martínez

**Affiliations:** 1 MOSABI Research Group, Physiotherapy Program, Faculty of Health Sciences, Universidad del Sinú Elías Bechara Zainúm, Montería 230001, Colombia; leilymontoya@unisinu.edu.co; 2 ÉNFISIS Research Group, Physiotherapy Program, Faculty of Health Sciences, Universidad del Sinú Elías Bechara Zainúm, Montería 230001, Colombia; ferminavasquez@unisinu.edu.co; 3 Physiotherapy Program, Faculty of Health Sciences, Universidad del Sinú Elías Bechara Zainúm, Montería 230001, Colombia; edgarfrodriguez@unisinu.edu.co (E.R.-S.); liztperezp@gmail.com (L.P.); sanchezperezanamarlen@gmail.com (A.S.P.); echeverrisergey@gmail.com (S.C.E.M.)

**Keywords:** older adults, virtual reality, exercise therapy, functional capacity, pilot intervention study

## Abstract

**Highlights:**

**What are the main findings?**

- A virtual reality-assisted exercise program provided preliminary descriptive information on potential favorable changes in cardiovascular health and functional capacity among institutionalized older adults.
- Participants demonstrated 100% adherence to the intervention, completing all prescribed sessions (10/10), and reported high satisfaction, with no adverse events during the intervention.

**What are the implications of the main findings?**

- Virtual reality appears to be a feasible strategy to enhance engagement and adherence in exercise programs for older adults in institutional settings.
- These preliminary findings support further exploration of immersive technologies in rehabilitation and health promotion programs for older adults.

**Abstract:**

Background: Physical inactivity and functional decline are common among institutionalized older adults, increasing the risk of cardiovascular disease and loss of independence. Innovative strategies such as virtual reality-assisted exercise may enhance adherence and improve health outcomes in this population. Methods: A pilot intervention study with a pre-post design was conducted in five institutionalized older adults who participated in a virtual reality-assisted multicomponent exercise program. The intervention included sessions combining strength, aerobics, balance, and flexibility exercises. Anthropometric, cardiovascular, and functional variables were assessed before and after the intervention. Physiological responses were assessed at the beginning of the sessions, midway through the intervention, and at the end of the program, along with participant satisfaction. Results: Preliminary descriptive changes were identified following the intervention, including lower values for body mass, body fat percentage, and systolic blood pressure, together with higher scores in several functional capacity measures, such as lower and upper body strength, aerobic endurance, and mobility. Physiological responses during exercise remained within expected ranges across sessions. All participants reported high satisfaction with the intervention, and no adverse events were documented. Conclusions: A multicomponent exercise program integrating virtual reality appears to be a feasible and well-tolerated intervention for institutionalized older adults. The preliminary descriptive findings suggest potential favorable changes in cardiovascular health and functional capacity; however, because no control group was included, the specific contribution of the virtual reality component cannot be determined.

## 1. Introduction

Aging is a global phenomenon associated with progressive declines in physical capacity, functional independence, and overall quality of life. Regular physical activity is recognized as a key strategy to preserve functional capacity and reduce the risk of chronic diseases in older adults [1,2]. Current international guidelines recommend at least 150–300 min of moderate-intensity physical activity per week for adults, including older populations, to maintain health and functional capacity [2].

Institutionalized older adults represent a particularly vulnerable population, often characterized by lower levels of physical activity, higher prevalence of chronic conditions, and increased risk of functional decline. Factors such as sedentary behavior and reduced environmental stimulation contribute to the deterioration of physical function, negatively impacting autonomy and quality of life [3].

In recent years, virtual reality (VR) has emerged as an innovative tool in rehabilitation and physical training. VR systems create immersive environments that integrate visual, auditory, and kinesthetic stimuli, allowing users to interact with simulated scenarios that may enhance engagement and motivation during exercise [4]. This is especially relevant in institutionalized settings, where access to stimulating and varied environments is often limited.

Recent systematic reviews and meta-analyses have demonstrated that VR-based interventions can improve balance, gait, and overall physical function in older adults when compared to conventional or minimal interventions [5,6]. Additionally, VR-assisted exercise has been associated with improvements in both physical and cognitive performance, highlighting its potential as a multidimensional therapeutic strategy [7].

Despite these promising findings, the current evidence remains heterogeneous, with variations in intervention protocols, duration, and study populations. However, most VR-based exercise studies have been conducted in community-dwelling older adults, and evidence from institutionalized populations remains scarce. In particular, few studies have examined the feasibility and implementation of short-term VR-assisted exercise programs in long-term care settings, where comorbidities, frailty, and functional limitations may substantially influence participation and outcomes [6].

Therefore, the aim of this pilot intervention study was to explore the feasibility and describe preliminary changes in physical capacity, cardiovascular parameters, and exercise response following the implementation of a VR-assisted exercise program in institutionalized older adults.

## 2. Materials and Methods

### 2.1. Study Design

A prospective pilot intervention study with a pre-post design was conducted to explore the feasibility of implementing a VR-assisted exercise program and to describe intra-individual changes among institutionalized older adults.

### 2.2. Participants

The study was conducted in a long-term care institution in Montería, Colombia. Participants were recruited using a convenience sampling approach among residents who were available and met the eligibility criteria during the study period.

A total of five older adults were included in the study.

Inclusion criteria were age ≥ 65 years, residence in the long-term care institution during the study period, ability to understand and follow simple instructions, sufficient functional capacity to participate in supervised physical activity, and ability to use the virtual reality device under professional supervision.

Exclusion criteria included medical conditions contraindicating exercise participation, advanced neurodegenerative disorders, significant visual or auditory impairments, or symptoms such as vertigo that could interfere with safe participation.

**Figure 1:**
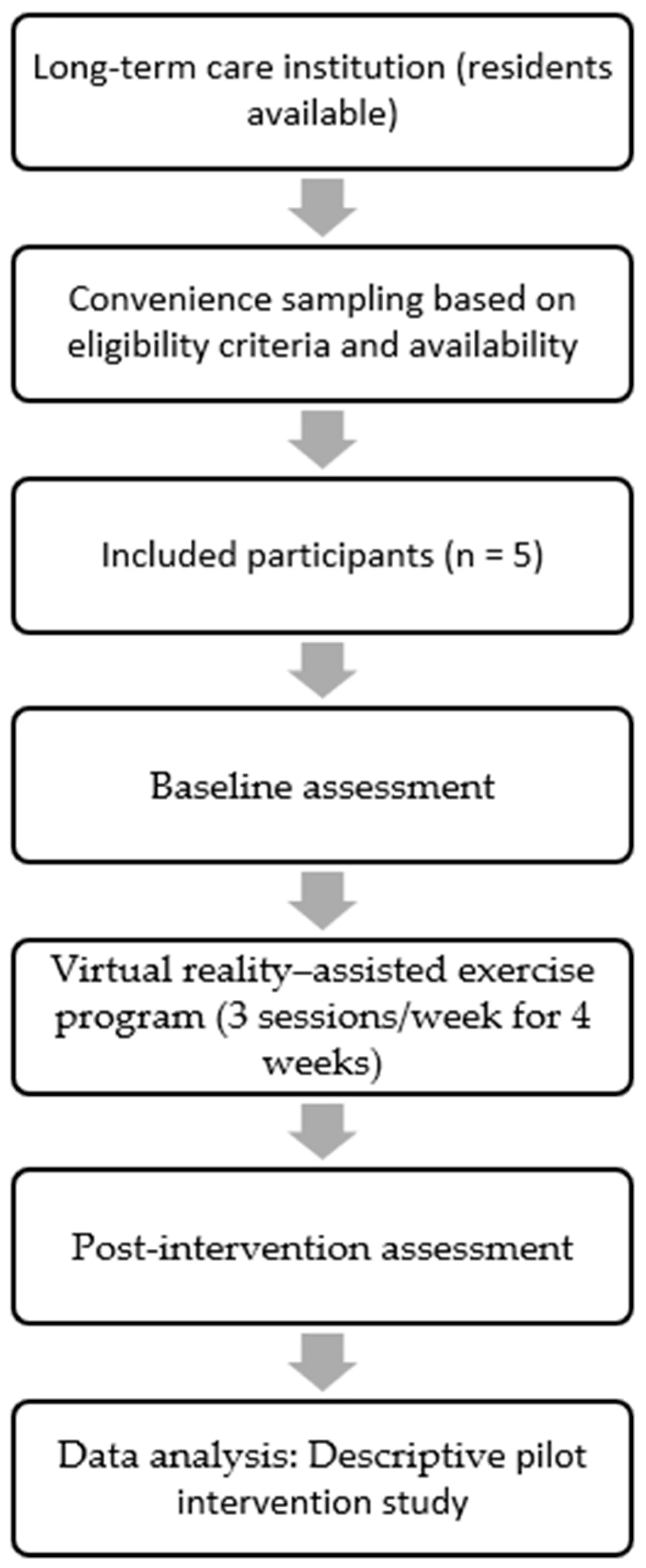
Flow diagram of participant selection, intervention, and analysis.

All participants provided written informed consent prior to inclusion. The participant selection and study process are illustrated in Figure 1.

### 2.3. Intervention

Participants completed a VR-assisted exercise program over four weeks, consisting of 10 sessions. Each complete session lasted approximately 60 min and included a warm-up period (5–10 min), a structured VR-assisted exercise phase with intermittent recovery periods, and a cool-down period (5–10 min).

Active exercise exposure time corresponded only to the main intervention phase and did not represent the total duration of each session. This exposure time varied according to the number and complexity of exercises performed in each session. Virtual Reality System and Exercise Protocol.

The intervention was delivered using the Oculus Quest 2 (Meta Platforms, Menlo Park, CA, USA), a standalone head-mounted display operating on Meta Horizon OS, which is based on the Android operating system. Participants performed the exercise sessions within an immersive virtual environment provided through the Meta ecosystem. The intervention was supervised throughout the study period by a licensed physiotherapist from the research team.

The VR-assisted training consisted of interactive exercise tasks integrated into a multicomponent physical exercise program. The training included cardiovascular activities, lower- and upper-limb strengthening tasks, balance and postural control activities, coordination exercises, and combined strength–cardiovascular tasks. Within the immersive virtual environment, participants were encouraged to perform functional movements such as reaching, stepping, weight shifting, arm movements, trunk control tasks, and dynamic balance activities according to their individual capacity. The VR environment was used to increase engagement and active participation during the prescribed exercises, rather than as an isolated intervention.

Exercise intensity progressed gradually across sessions according to each participant’s individual tolerance and physiological response. Progression was based on the ability to complete the prescribed movements safely, perceived exertion using the Borg scale, and heart rate and blood pressure (BP) responses recorded during the sessions. When participants tolerated the session without excessive fatigue, adverse symptoms, or abnormal cardiovascular responses, the exercise load was adjusted by increasing the duration of active exercise exposure, the number of exercises, or the complexity of the motor tasks. Conversely, when fatigue, discomfort, elevated perceived exertion, or atypical physiological responses were observed, the exercise load was maintained or reduced, and additional recovery time was provided.

All intervention sessions were supervised by a licensed physiotherapist from the research team, who was trained in therapeutic exercise and monitoring of older adults during physical activity. The supervising physiotherapist was responsible for ensuring correct execution of the exercises, monitoring participants before, during, and after exercise, identifying symptoms such as dizziness, fatigue, discomfort, or visual intolerance, adjusting exercise intensity when necessary, and ensuring the safe use of the VR device. Safety procedures included checking participant tolerance to the head-mounted display and providing seated or supported alternatives when needed. Adherence was monitored by recording attendance and completion of each session for every participant throughout the intervention period.

### 2.4. Outcome Measures

Assessments were performed before and after the intervention.

The variables assessed, measurement instruments, and their clinical relevance are summarized in Table 1.

Anthropometric and body composition variables included body mass, body mass index (BMI), body fat percentage, muscle mass percentage, waist circumference, hip circumference, and waist-to-hip ratio.

Cardiovascular parameters included systolic and diastolic BP and heart rate.

Anthropometric, body composition, cardiovascular, and physiological measurements were obtained using standardized procedures. Body mass was measured using a commercial digital scale sourced in Montería, Colombia; however, the specific brand and model were not available. Body composition variables, including body fat percentage and muscle mass percentage, were estimated using an Omron HBF-514C body composition monitor (Omron healthcare, Inc.), acquired in Montería, Colombia, in 2019. This device uses bioelectrical impedance analysis to estimate body composition parameters. Waist and hip circumferences were measured using a flexible tailoring measuring tape sourced in Montería, Colombia; however, the specific brand and manufacturer information were not available. Blood pressure was assessed using a manual aneroid sphygmomanometer with an adult cuff, sourced in Montería, Colombia; however, the specific brand, model, and manufacturer information were not available.Heart rate was monitored using a Lifen pulse oximeter sourced in Montería, Colombia; however, the specific manufacturer information was not available. All measurements were performed by trained personnel using the same procedures at baseline and post-intervention. Given that bioelectrical impedance analysis provides indirect estimates of body composition, these results should be interpreted cautiously within the exploratory context of this pilot intervention study.

Functional capacity was assessed using the Senior Fitness Test battery, including the chair stand test, arm curl test, 2 min step test, chair sit-and-reach test, back scratch test, and the Timed Up and Go test.

Additionally, participants’ physiological and perceptual responses during exercise sessions were monitored using heart rate, blood pressure, and the Borg Rating of Perceived Exertion scale.

Participant satisfaction was assessed after completion of the intervention using a single-item Likert-type measure: “Overall satisfaction with the virtual reality-assisted exercise program.” Responses were rated on a 5-point scale ranging from 1 (very dissatisfied) to 5 (very satisfied). This item was used as a simple indicator of acceptability within the feasibility-oriented design of the pilot intervention study and was not intended to represent a validated multidimensional satisfaction questionnaire.

Given the exploratory nature of this pilot study, small variations in anthropometric measures (e.g., waist-to-hip ratio) should be interpreted cautiously, as they may fall within the expected range of measurement errors.

### 2.5. Data Collection Procedures

Baseline assessments included sociodemographic and health-related variables, followed by physical and functional evaluations. Post-intervention assessments included the same measurements.

Physiological responses were recorded at different time points during selected sessions (rest, during exercise, and recovery) to monitor safety and tolerance to the intervention.

### 2.6. Data Analysis

Data were analyzed using a descriptive approach. Continuous variables are presented as means and standard deviations, and individual pre–post changes were examined.

Given the small sample size and the descriptive nature of the study, no inferential statistical analysis was performed. Emphasis was placed on describing intra-individual variability and observed trends across participants.

### 2.7. Ethical Considerations

The study was conducted in accordance with the principles of the Declaration of Helsinki. The research protocol was reviewed and endorsed by the Research Committee of the Faculty of Health Sciences at Universidad del Sinú, which acted as the faculty-level institutional body responsible for the methodological and ethical review of research projects within the Faculty of Health Sciences, as recorded in Act No. 002 of 2024, dated 22 March 2024, under item 9.d. According to national regulations for health research in Colombia (Ministry of Health of Colombia, Resolution 8430 of 1993), the study was classified as minimal risk, as it involved a supervised exercise intervention without invasive procedures [8]. All participants provided written informed consent prior to their inclusion in the study. All procedures were conducted under the supervision of a licensed physiotherapist from the research team to ensure participant safety, and no adverse events were reported during the intervention.

## 3. Results

A total of five institutionalized older adults participated in the study. The sample included four men and one woman, with a mean age of 74.8 years.

### 3.1. Baseline Characteristics and Health Habits

Baseline characteristics, medical conditions, and lifestyle habits of the participants are presented in Table 2.

None of the participants reported current tobacco use. Regarding alcohol consumption during the previous 12 months, 2 out of 5 participants reported alcohol intake, 2 denied consumption, and information was unavailable for 1 participant because this item was not fully reported during baseline data collection.

All participants reported daily consumption of fruits and vegetables; however, the quantity consumed was limited. Three out of five participants reported consuming only one serving of fruit per day, whereas three participants reported consuming two servings of vegetables daily.

In terms of clinical conditions, elevated BP was present in 2 out of 5 participants, whereas diabetes and hypercholesterolemia were each reported by 1 participant, indicating the presence of cardiovascular risk factors within the sample.

### 3.2. Exercise Program Description

The characteristics of the exercise prescription across sessions are summarized in Table 3.

The intervention program was structured into sessions that combined cardiovascular, strength, balance, and coordination exercises, with variability in the number of exercises, duration, and recovery time across sessions.

### 3.3. Physiological Response to Exercise

Physiological responses during selected intervention sessions (Sessions 1, 5, and 10) are presented in Table 4.

Across these sessions, perceived exertion ranged from low to moderate values according to the Borg scale, with scores between 5.5 and 6.8 during the main exercise phase.

Heart rate increased during the active phases of exercise and returned toward baseline values during recovery across all evaluated sessions, remaining within expected physiological ranges for older adults.

BP showed the expected acute response pattern to physical activity, with increases during exercise and a return toward resting levels during recovery. This pattern was consistent across sessions 1, 5, and 10.

One participant presented higher BP values at rest and during exercise, consistent with a previous diagnosis of hypertension. However, inclusion of this participant did not modify the overall physiological response patterns observed.

### 3.4. Changes in Anthropometric, Cardiovascular, and Functional Variables

Pre- and post-intervention outcomes are presented in Table 5.

To improve transparency and facilitate the interpretation of individual variability, participant-level pre- and post-intervention data are provided in Supplementary Table S1.

Descriptive pre–post comparisons suggested lower values for body mass, BMI, and body fat percentage following the intervention, whereas muscle mass showed a slight increase. Waist circumference remained relatively stable, while hip circumference showed a small reduction, resulting in minimal changes in the waist-to-hip ratio. Similarly, systolic and diastolic BP values appeared lower after the intervention, whereas heart rate showed a slight increase compared with baseline measurements.

Functional performance measures also showed descriptive improvements across several domains. Higher scores were observed in lower- and upper-body strength, as assessed by the chair stand and arm curl tests, respectively, while aerobic endurance appeared to increase according to the 2 min step test. Modest positive changes were also identified in lower and upper body flexibility, and mobility and balance appeared to improve, as reflected by shorter completion times in the Timed Up and Go test. Given the exploratory nature of this pilot intervention study and its limited sample size, these findings should be interpreted as preliminary observations.

### 3.5. Participant Experience

Participant satisfaction with the VR-assisted intervention was assessed after completion of the program using a single-item Likert-type measure: “Overall satisfaction with the VR-assisted exercise program.” All participants assigned the maximum score of 5, indicating very high overall satisfaction with the intervention.

Participants consistently described the intervention as engaging and well-tolerated, highlighting the immersive characteristics of the virtual environments as a factor that facilitated adherence and participation during the exercise sessions. No adverse events or discomfort associated with the use of VR were reported.

## 4. Discussion

The present pilot intervention study explored the feasibility and preliminary descriptive changes associated with a multicomponent exercise program integrating VR in institutionalized older adults. Overall, the findings suggest that this approach is feasible and well tolerated, with descriptive changes observed in systolic BP and functional capacity, together with high participant satisfaction.

The small descriptive changes observed in body mass, body fat percentage, and BMI should be interpreted with caution, given the exploratory nature of this pilot intervention study, the short duration of the program, and the limited sample size. Although structured exercise interventions may influence body composition in older adults, these adaptations usually require longer intervention periods and should not be inferred from the present findings.

From a cardiovascular perspective, the reduction in systolic BP represents a clinically relevant descriptive finding. Exercise-based interventions have been shown to be effective non-pharmacological strategies for BP control in older adults [9,10]. These effects are particularly relevant given the baseline presence of cardiovascular risk factors in the study population. The variability observed in diastolic BP may be explained by individual clinical differences and the small sample size inherent to pilot study designs. The slight increase observed in resting heart rate should also be interpreted cautiously. Given the very small sample size, this descriptive change may reflect normal intra-individual variability or differences in measurement conditions, such as hydration status, emotional state, medication use, or timing of assessment, rather than a clinically meaningful physiological adaptation. Therefore, this finding should not be interpreted as evidence of the cardiovascular effect of the intervention.

Improvements in functional capacity across strength, aerobic endurance, and mobility domains are consistent with evidence supporting multicomponent exercise interventions for improving physical function and reducing frailty risk in older adults [11,12]. In addition, the observed improvements in strength-related tests are in line with studies demonstrating the benefits of resistance training in older adults with chronic conditions [13]. Considering that institutionalized older adults often present lower baseline levels of physical function, these descriptive changes may be clinically relevant; however, they should be interpreted cautiously due to the exploratory nature of the study and the absence of a control group [3].

The integration of VR into the exercise program may have contributed to the high levels of adherence and satisfaction reported by participants, although the absence of a comparison group prevents isolating the specific contribution of the VR component. All individuals reported maximum satisfaction scores, and no adverse events were identified, supporting the feasibility and short-term safety of this approach. Recent evidence suggests that VR-based interventions can enhance motivation, engagement, and adherence in older adults by providing immersive and interactive environments [6,14]. Although much of the evidence has been generated in clinical populations, such as individuals with neurological conditions, similar benefits have been reported in rehabilitation contexts, supporting the broader applicability of this technology [5].

When compared with previous studies conducted in institutionalized or frail older adults, the present findings are consistent with the growing evidence suggesting that VR-based exercise may be feasible and acceptable in populations with greater vulnerability. Campo-Prieto et al. reported that an immersive VR exergame program was feasible in institutionalized older adults and may contribute to changes in physical function, although the authors also emphasized the need for cautious interpretation due to sample size and implementation-related factors [15]. Similarly, studies involving frail or prefrail older adults have suggested that VR-based rehabilitation or exercise programs may support balance, mobility, engagement, and adherence; however, their effects on strength, functional mobility, and long-term clinical outcomes remain inconclusive [16]. In this context, the high satisfaction, absence of adverse events, and descriptive functional changes observed in the present pilot intervention study are aligned with prior evidence, while also reinforcing the need for larger controlled trials specifically in institutionalized older adults.

Despite these preliminary findings, several limitations must be acknowledged. The small sample size and the use of convenience sampling limit the generalizability of the results and preclude statistical inference. Additionally, the absence of a control group prevents causal interpretation of the observed changes. Because all participants received a multicomponent exercise program integrating VR, it is not possible to determine whether the descriptive changes identified were attributable to the exercise intervention itself, the VR component, or the combination of both. Furthermore, the relatively short duration of the program may have limited the magnitude of the observed changes, particularly in anthropometric variables. In addition, participant satisfaction was assessed using a single-item Likert-type measure rather than a validated multidimensional satisfaction questionnaire, which limits the depth of interpretation of acceptability and user experience. Therefore, these findings should be interpreted as exploratory and hypothesis-generating.

Future research should aim to include larger samples, longer intervention periods, and controlled designs to better establish the effectiveness of VR-assisted exercise programs in older adults. Additionally, further studies exploring long-term adherence, functional independence, and quality of life outcomes would provide valuable insights into the sustainability and clinical relevance of these interventions.

## 5. Conclusions

The findings of this pilot intervention study suggest that a multicomponent exercise program integrating VR is feasible and well-tolerated among institutionalized older adults. The descriptive pre–post changes observed in anthropometric, cardiovascular, and functional variables provide preliminary information that may guide future research. However, given the very small sample size, the absence of a control group, and the exploratory nature of the study, these findings should not be interpreted as evidence of clinical effectiveness. In addition, the specific contribution of the VR component cannot be isolated from the effects of the exercise intervention itself. Larger controlled studies are needed to determine the effectiveness, safety, and clinical applicability of VR-assisted exercise interventions in this population.

## Figures and Tables

**Table 1:** Outcome measures, instruments, and clinical relevance.

Variable Category	Variable	Instrument/Method	Measurement Unit	Purpose/Clinical Relevance
Anthropometric	Body mass	Commercial digital scale; brand/model not available	kg	Assessment of overall body weight changes
Anthropometric	BMI	Commercial digital scale; brand/model not available	kg/m^2^	Classification of nutritional status
Body composition	Body fat percentage	Omron HBF-514C body composition monitor; bioelectrical impedance analysis	%	Estimation of adiposity levels
Body composition	Muscle mass percentage	Omron HBF-514C body composition monitor; bioelectrical impedance analysis	%	Evaluation of muscle mass changes
Anthropometric	Waist circumference	Flexible tailoring measuring tape	cm	Indicator of central adiposity
Anthropometric	Hip circumference	Flexible tailoring measuring tape	cm	Assessment of body fat distribution
Anthropometric	Waist-to-hip ratio	Calculated	Ratio	Cardiometabolic risk indicator
Cardiovascular	Systolic blood pressure	Manual aneroid sphygmomanometer with adult cuff	mmHg	Assessment of cardiovascular risk
Cardiovascular	Diastolic blood pressure	Manual aneroid sphygmomanometer with adult cuff	mmHg	Assessment of cardiovascular regulation
Cardiovascular	Heart rate	Lifen pulse oximeter	bpm	Monitoring of physiological response to exercise
Functional capacity	Lower body strength	Chair stand test	Repetitions	Assessment of lower limb strength and functional independence
Functional capacity	Upper body strength	Arm curl test	Repetitions	Assessment of upper limb strength
Functional capacity	Aerobic endurance	2 min step test	Steps	Evaluation of cardiorespiratory endurance
Functional capacity	Lower body flexibility	Chair sit-and-reach test	cm	Assessment of hamstring and lower back flexibility
Functional capacity	Upper body flexibility	Back scratch test	cm	Assessment of shoulder flexibility
Functional capacity	Mobility and balance	Timed Up and Go test	seconds	Evaluation of mobility, balance, and fall risk
Exercise response	Perceived exertion	Borg scale	0–10 scale	Subjective assessment of exercise intensity

Abbreviations: BMI, body mass index; bpm, beats per minute. Measurements were obtained using standardized procedures. Functional capacity was assessed using the Senior Fitness Test battery. Waist-to-hip ratio was calculated as waist circumference divided by hip circumference. All measurements were performed by trained personnel.

**Table 2:** Baseline characteristics, health conditions, and lifestyle habits of participants.

Variable	Category	*n*
Current tobacco use	Yes	0
	No	5
Alcohol consumption (last 12 months)	Yes	2
	No	2
	No information available	1
Fruit intake (servings/day)	1	3
	2	1
	3	1
Vegetable intake (servings/day)	2	3
	3	2
Elevated blood pressure	Yes	2
	No	3
Diabetes or high blood sugar	Yes	1
	No	4
High cholesterol	Yes	1
	No	4

Abbreviations: *n*, number of participants. Values are presented as absolute frequencies. “No information available” indicates missing or unreported self-reported information during baseline data collection.

**Table 3:** Characteristics of the exercise program across intervention sessions.

Session	Number of Exercises	Active Exercise Time (min)	Recovery Time (min)	Main Exercise Type
1	3	22.0	3.75	Cardiovascular
2	4	25.3	3.25	Balance/Strength
3	5	30.0	5.25	Strength
4	5	22.0	5.50	Strength/Cardio
5	5	11.0	6.42	Strength
6	6	19.0	7.00	Strength/Cardio
7	4	17.5	4.50	Balance/Strength
8	5	31.0	7.50	Strength
9	5	30.0	6.75	Strength/Cardio
10	5	11.0	4.17	Strength/Cardio

Note: Values represent active exercise exposure time during the main intervention phase and do not include warm-up or cool-down periods. Each complete session lasted approximately 60 min, including warm-up, VR-assisted exercise activities, intermittent recovery periods, and cool-down. Recovery time refers to intermittent rest periods between exercises within each session.

**Table 4:** Physiological responses during selected intervention sessions (Sessions 1, 5, and 10).

Session	Time Point	Systolic BP (mmHg)	Diastolic BP (mmHg)	Heart Rate (bpm)	Perceived Exertion (Borg)
Session 1	Rest	114	74	78	5.8
	15 min	118	84	84	
	30 min	120	76	86	
	45 min	114	82	82	
	Recovery	112	76	71	
Session 5	Rest	106	76	80	5.6
	15 min	114	84	86	
	30 min	118	76	81	
	45 min	122	72	79	
	Recovery	110	72	77	
Session 10	Rest	108	72	78	6.8
	15 min	122	80	89	
	30 min	124	80	93	
	45 min	116	78	84	
	Recovery	112	70	74	

Note: Values represent mean responses across participants. BP: blood pressure; bpm: beats per minute. Perceived exertion was assessed using the Borg scale during the main phase of exercise.

**Table 5:** Changes in anthropometric, cardiovascular, and functional variables before and after the intervention.

Variable	Baseline (Mean ± SD)	Post-Intervention (Mean ± SD)	Mean Change ± SD
Anthropometric variables			
Body mass (kg)	71.5 ± 10.3	70.14 ± 11.19	1.36 ± 0.91
BMI (kg/m^2^)	24.92 ± 4.47	24.48 ± 4.88	0.44 ± 0.31
Body fat (%)	29.98 ± 10.59	27.57 ± 10.22	2.41 ± 1.45
Muscle mass (%)	34.77 ± 9.33	35.28 ± 9.54	−0.51 ± 0.47
Waist circumference (cm)	95.8 ± 13.54	95.4 ± 12.84	0.40 ± 0.93
Hip circumference (cm)	107.6 ± 8.3	105.4 ± 9.21	2.20 ± 1.77
Waist-to-hip ratio	0.89 ± 0.07	0.90 ± 0.05	−0.01 ± 0.01
Cardiovascular variables			
Systolic BP (mmHg)	121.4 ± 9.76	109 ± 8.34	12.4 ± 3.76
Diastolic BP (mmHg)	79.2 ± 9.94	70.4 ± 8.85	8.8 ± 5.51
Heart rate (bpm)	71 ± 10.89	74 ± 13.15	−3.0 ± 3.49
Functional capacity variables			
Chair stand (repetitions)	9.6 ± 2.19	13 ± 3.94	−3.4 ± 0.93
Arm curl (repetitions)	13.4 ± 4.67	16.4 ± 4.04	−3.0 ± 0.45
2 min step test (steps)	46.6 ± 20.62	71 ± 16	−24.4 ± 11.62
Chair sit-and-reach (cm)	0.85 ± 0.80	1.0 ± 1.51	−0.15 ± 1.22
Back scratch (cm)	−17.25 ± 12.35	−13.65 ± 11.35	−3.6 ± 1.39
Timed Up and Go (s)	13.91 ± 2.26	11.63 ± 4.03	2.27 ± 1.36

Note: Values are presented as mean ± standard deviation. Mean change was calculated as baseline minus post-intervention values; therefore, positive values indicate reductions and negative values indicate increases.

## Data Availability

The data presented in this study are available upon reasonable request from the corresponding author. The datasets are not publicly available due to ethical and privacy restrictions, as they contain sensitive personal information of the older adults who participated in the study.

## Supplementary Materials

The following supporting information can be downloaded at: https://www.mdpi.com/article/10.3390/healthcare14162472/s1. Supplementary Table S1. Individual participant-level data before and after the virtual reality-assisted exercise program, including anthropometric, cardiovascular, and functional capacity variables.

## Abbreviations

The following abbreviations are used in this manuscript:

VR	Virtual Reality
BMI	Body Mass Index
BP	Blood Pressure
