# Supplementary material for: Physical Capacity Changes Following a Virtual Reality-Assisted Exercise Program in Institutionalized Older Adults: A Pilot Intervention Study
Source: Healthcare (Basel). 2026 Aug 10;14(16):2472. doi: 10.3390/healthcare14162472 (PMC13512789; doi:10.3390/healthcare14162472)
Supplement: Supplementary file 1 [file healthcare-14-02472-s001.zip › healthcare-4324730-supplementary.pdf]

Supplementary Table S1. Individual participant-level data before and after the virtual reality-assisted exercise program, including anthropometric, cardiovascular, and functional capacity variables .

Variable	P1 Pre	P1 Post	P2 Pre	P2 Post	P3 Pre	P3 Post	P4 Pre	P4 Post	P5 Pre	P5 Post
**Anthropometric variables**										
**Body mass (kg)**	70	68	71.2	67	88	89.2	59.6	59.5	68.7	67
**BMI (kg/m²)**	21.6	21.1	21.9	20.6	31.6	32	22	21.9	27.6	26.8
**Body fat (%)**	31.03	28.07	32.08	24.8	30.7	32.2	13.3	12.5	42.8	40.3
**Muscle mass (%)**	48.27	49	33	35	30.3	29.4	38.8	39	23.5	24
**Waist circumference (cm)**	90	91	92	90	116	116	80	82	101	98
**Hip circumference (cm)**	109	100	99	98	119	120	100	100	111	109
**Waist-to-hip ratio**	0.83	0.91	0.93	0.92	0.97	0.97	0.80	0.82	0.91	0.90
**Cardiovascular variables**										
**Systolic BP (mmHg)**	120	110	110	110	130	130	130	110	110	100
**Diastolic BP (mmHg)**	90	70	80	80	90	80	80	50	70	70
**Heart rate (bpm)**	67	88	62	64	89	87	61	57	75	75
**Functional capacity variables**										
**Chair stand (repetitions)**	8	10	8	11	12	15	12	19	8	10
**Arm curl (repetitions)**	6	10	12	15	16	18	18	20	15	19
**2-minute step test (steps)**	15	48	42	46	47	70	69	86	60	69
**Chair sit-and-reach (cm)**	2	1	1.5	2	0	0	1.5	3.5	0	0
**Back scratch (cm)**	-8	-7	-30	-22	-4	-2	-12.5	-5.5	-31	-29
**Timed Up and Go (s)**	15.6	15.25	13.05	12.16	10	8.5	13.05	6.7	17.03	16.01
